# Immunohistochemical localization and mRNA quantification of osteopontin and Tamm-Horsfall protein in canine renal tissue after potassium oxalate injection

**DOI:** 10.1186/1746-6148-10-70

**Published:** 2014-03-17

**Authors:** Walaa Mohamaden, Heng Wang, Huawei Guan, Xia Meng, Jianji Li

**Affiliations:** 1 Department of Clinical Veterinary Medicine, College of Veterinary Medicine, Yangzhou University, Yangzhou, Jiangsu 225009, China; 2 Jiangsu Co-innovation Center for Prevention and Control of Important Animal Infectious Diseases and Zoonoses, Yangzhou, Jiangsu 225009, China

**Keywords:** Canine, Osteopontin, Tamm-Horsfall, mRNA, IHC

## Abstract

**Background:**

Urinary macromolecules contribute to promoting or inhibiting crystal retention in renal tissue and stone formation. Osteopontin (OPN) and Tamm-Horsfall protein (THP) are the most important proteins involved in this process. Although these two proteins were discovered a long time ago, their role in setting kidney stone formation has not yet been fully investigated. We conducted a study to explore the role of OPN and THP in canine renal oxalosis. Ten dogs were carefully examined prior to the study. Six dogs were assigned to the treatment group and were injected intravenously with 0.5 M potassium oxalate (KOx). The other four dogs were assigned to a control group and were injected intravenously with 0.9% NaCl three times a day (tid) for 7 consecutive days. Then kidneys were harvested for pathological, immunohistochemical examination and OPN and THP mRNA expression levels were quantified by quantitative real-time PCR.

**Results:**

Calcium oxalate crystals deposition was observed in both renal cortex and medulla. Immunohistochemistry examination revealed increased tissue expression of OPN in the renal tissue while THP was significantly decreased. OPN mRNA expression level significantly increased in treated dogs compared to that in the controls, while THP mRNA level significantly decreased.

**Conclusion:**

Together, these results suggest that THP and OPN are both involved in the pathogenesis and response to oxalate exposure.

## Background

In the past two decades, calcium oxalate urolithiasis (COU) has been a frustrating problem in dogs and cats worldwide [1]. Usually the presence of oxalate crystals in urine is a result from urinary supersaturation of oxalate salts. A problem that might be established in kidneys first and subsequently leads to retention of oxalate crystals within the renal tissue, inducing injury to renal tubules [2] due to adhesion between crystals and renal epithelial tissue [3]. Kidneys normally produce macromolecules such as osteopontin (OPN), nephrocalcin, fibronectin, Tamm-Horsfall protein (THP) and other macromolecules that bind to calcium salts in order to prevent or promote crystal growth and aggregation [4].

OPN is a negatively-charged aspartic acid-rich protein expressed in tissues, such as bone, liver, kidney, lung, bladder, and pancreas, as well as in macrophages [5]. It inhibits the formation of calcium oxalate (CaOx) crystals and regulates physiologic and pathologic mineralization [6]. The role of OPN in nephrolithiasis formation is unclear*. In vitro,* studies suggested that OPN inhibits nucleation, and CaOx crystal growth and aggregation in cultured epithelial cells. It also enhances the formation of calcium oxalate dihydrate (COD) crystals that are less adherent to renal tissue than calcium oxalate monohydrate (COM) crystals [7]. Conversely, other studies found OPN associated with nephrolithiasis through the mineralization and dystrophic calcification of the urolithiasis matrix [8]. OPN is also an important mediator of tissue injury. Increased expression of OPN in renal tubule cells is related to an accumulation of macrophages in the damaged tissue, since OPN is involved in the recruitment and retention of macrophages in the inflamed site [9-11].

Another urinary macromolecule involved in renal tissue crystals deposition is uromodulin or THP, a glycosylphosphatidylinositol protein, the most abundant urinary protein and the main constituent of hyaline urinary casts [12]. THP forms a gel like matrix that traps bacteria and prevents their adhesion to plasma membranes. It also acts as an inhibitor of stone formation in healthy individuals by trapping crystals in the same manner [13]. However, this function may be subverted under some circumstances and THP may facilitate crystal aggregation and then promote stone formation [14]. In humans, studies investigating the association between THP expression and urinary excretion were inconsistent in stone forming patients [15]. THP was expressed in the thick ascending limbs of Henle’s loops in normal renal tissue of dogs [16], and it was chosen as a new biomarker of canine renal toxicity [17], indicating that THP is involved in the development of renal diseases in dogs.

Despite numerous proteins that engaged in the retention of CaOx crystals in the urinary tract, understanding the precise mechanisms by which OPN and THP influence the several aspects of crystallization and urinary stone formation is only beginning to emerge. For instance, THP expression decreased in ethylene glycol-administered rats [18], while in other studies it increased [19] or remained unchanged in others [20]. Some studies dealt only with one of the two proteins, this does not provide a logical understanding of how these two proteins interact. Most of the studies that focused on both proteins were conducted in either laboratory animals [18,21] or in Madin Darby canine kidney cell culture (MDCK) [22], which may not be representative of the real situation in canine renal tissue *in vivo.* However the conflicting roles of THP and OPN makes the study of their reaction toward kidney stone formation also valuable for the explanation of the pathogenesis of renal oxalosis in dogs. We hypothesized that THP and OPN expression in the renal tissue will be changed after exposure to oxalates. The aim of current study is to record the canine renal tissue injury upon oxalates injection *in vivo* and to quantify THP and OPN gene expressions of renal tubular cells in order to investigate their role of pathogenesis in canine renal oxalosis.

## Methods

### Chemicals

0.5 mol/l KOx (K_2_C_2_O_4_ · H_2_O) (MW = 184 g) was prepared KOx solution was filtered (0.22 μm) prior to injection and transfused to dogs in the treatment group at a dose of 0.13 ml/kg. The control group received the same volume of physiological saline.

### Design

Ten experimentally-naive healthy intact adult beagles over the age range of 2.5-3 years and body weight ranged between 8-12 kg were provided from the research unit of the Department of Clinical Veterinary Medicine, College of Veterinary Medicine, Yangzhou University. Dogs were randomly assigned to the treatment group (n = 6, four males and two females) and control group (n = 4, one male and three females). Dogs were housed separately in stainless steel cages. All dogs were fed commercial diet twice a day and water was provided *ad libitum* for acclimatization. All dogs received clinical examinations including creatinine, urea nitrogen and urine specific gravity tests before conducting the experiment. None of the dogs had any previous history of long-term illness. Butterfly catheters were fixed in the cephalic veins. Each group was injected with the corresponding solution three times a day for seven days. Eight hours after the last injection, kidney samples were collected by surgical incision in the midabdomen under thiobarbiturate anesthesia (20 mg/kg). Dogs were then euthanized by injecting the lethal dose of the anesthetic agent. All experiments and procedures performed on the animals were approved by the Animal Care and Ethical Committee of Yangzhou University.

### Histologic and immunohistochemical examination

Kidney tissue was fixed by immersion in 4% paraformaldehyde in 0.1 M phosphate buffer saline (pH = 7.4), and embedded in paraffin. Four-micrometer thick sections were stained by H&E and Pizzolato’s method [23] to detect the presence of calcium oxalates. Deparaffinized tissue sections were incubated in 0.3% H_2_O_2_ in methanol for 10 min, followed by washing with distilled water, then followed by citrate buffer (pH = 6.0) antigen retrieval. Nonspecific binding was blocked with 3% bovine serum albumin for 30 min, and the slides were incubated with polyclonal rabbit anti-OPN or polyclonal rabbit anti-THP antibodies (Beijing Biosynthesis Biotechnology Company, China) at 4°C overnight. Slides were incubated 30 min at room temperature with horseradish peroxidase conjugated species-specific secondary antibody. Diaminobenzidine (DAB) was used as chromogen to detect OPN and THP signals. Then slides were counterstained with hematoxyline, dehydrated and mounted under coverslip. Negative control sections were incubated with phosphate buffer saline. Images were analyzed by an inverted light microscope equipped with a Quick Imaging System (Leica DM2500, Leica, Germany).

### RNA extraction and real-time quantitative reverse transcription PCR

After kidney collection, the kidney was divided into cortex and medulla, each part was cut into small parts of kidney tissues and directly dipped in RNA fixer (Takara, Cat9750) and stored at -20°C until RNA extraction. Total RNA was extracted using Trizol reagent (Trizol, RNAiso Plus, Takara, Dalian). RNA was eluted in nuclease-free water and subjected to DNase to remove genomic DNA (Recombinant DNASE I, Takara, Dalian). After DNase treatment, RNA was quantified with Nano Drop. The absorption ratio (OD260nm/OD280nm) was between 1.8 and 2.2, indicating a high quality RNA. Samples were immediately reverse-transcribed using 3 μg of extracted RNA per sample (Primescript 1st strand, Takara, Dalian). The resulting cDNA was stored at -20°C until analyzed. qPCR gene-specific primers for canine osteopontin (OPN, Accession Number: DQ195101.1) and canine Tamm-Horsfall protein (THP, Accession Number: AF498324.1) were designed and synthesized (Invitrogen, Shanghai, China). Ribosomal protein L13A (RPL13A, Accession Number: AJ388525) was used as a housekeeping gene, because it is the most stably expressed housekeeping gene in canine tissue [24] (Table 1).

**Table 1:** Primers for quantitative real-time PCR

**Gene**	**Forward primer**	**Reverse primer**	**Product size**
**OPN**	5-TAGCCAGGACTCCGTTGACT-3	5-ACACTATCACCTCGGCCATC-3	162
**THP**	5-TGCCTGGTGGGTTTCACT-3	5-CGAGTAATTGCCCTTGTTGTT-3	249
**RPL13A**	5-GCCGGAAGGTTGTAGTCGT-3	5-GGAGGAAGGCCAGGTAATTC-3	87

The cDNA was subjected to qPCR amplification using the SybrGreen (Sybrpremix EX Taq II, Takara, Dalian) in qPCR System (Applied Biosystems7500 Real-Time PCR System, Applied Biosystems, USA). qPCR was conducted in 20 μL of reaction agent composed of a water-base dilution of 10 μL SYBR Premix EX II, 2 μL cDNA templates, and 0.2 μL of each primer. Each sample was analyzed in triplicate. Thermal cycling conditions were identical for the 2 primers: 50°C for 2 min, 95°C for 30 s, 40 cycles at 95°C for 5 s, 60°C for 30 s, and 72°C for 30 s. Melting curve analysis identified a single PCR product after amplification of OPN, THP, and RPL13A. The fold change (n-fold) for gene expression was calculated using the relative quantification method (2^-ΔΔCt^).

### Statistical analysis

Student’s *t*-test using SPSS for Windows (Version 16.0, Chicago, SPSS Inc. Released in 2007) was used to compare the ΔCt values of treatment and control groups for both target genes separately. The level of significance at which the null hypothesis was rejected is α = 0.05. The statistical analyses were performed on the ΔCt values and processed into ΔΔCt and converted to n-fold (2^–ΔΔCt^) for data presentation.

## Results

### Histologic findings

**Figure 1:**
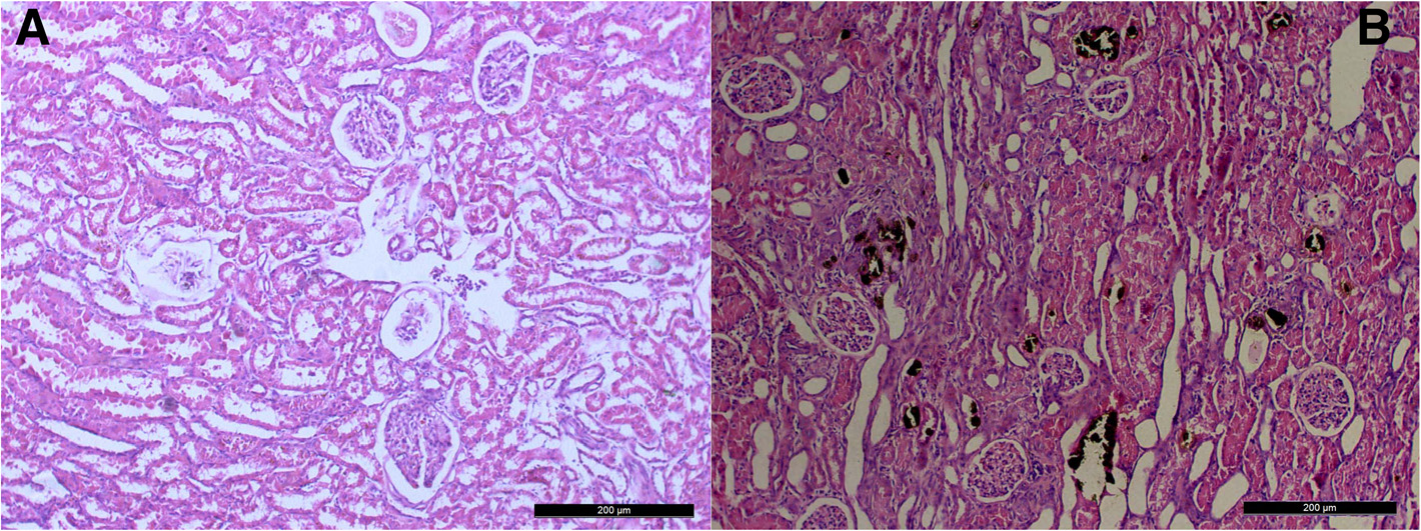
**Cross section in the renal tissue of both groups. A)** Control group. **B)** Treatment group, note the retention of CaOx crystals (black deposits) and tubular dilatation × 100. Pizzolatos and HE stain.

**Figure 2:**
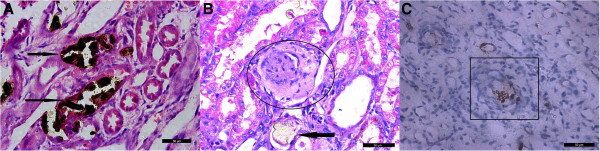
**Illustration to areas of inflammation in the renal tissue of treatment group. A)** CaOx crystals deposition (black arrows), tubular injury and inflammatory cells infiltration in the renal tissue, Pizzolatos and HE stains. **B)** Encircled area shows leukocytic infiltration, fibrin deposition and CaOx crystals retention (black arrow). HE stains. **C)** Square encloses the area of leukocytic infiltration and OPN expression. Antigens were detected using antibodies to osteopontin, followed by second antibodies coupled to horseradish peroxidase with DAB counterstained with hematoxelyne × 400.

Seven consecutive days of intravenous KOx injection resulted in intratubular retention of CaOx crystals. CaOx crystals were evident in both Pizzolato’s and H&E stained tissue sections. Crystals were present in both the renal medulla and cortex, and affected proximal nephrons, distal nephrons, collecting ducts, and some tubules appeared dilated, while there were no crystals observed in the renal tissue of control group as well as the renal tubules appeared normal and cells were intact (Figure 1). CaOx crystal induced injury to renal epithelium, loss of the brush border and apical membrane with leakage of the cellular content into the tubular lumen. Large amounts of leukocytes infiltration and fibrin deposition were observed in the renal tissue near crystals and in the interstitium in the cortex and medulla (Figure 2).

### Localization of OPN and THP in kidney tissue

**Figure 3:**
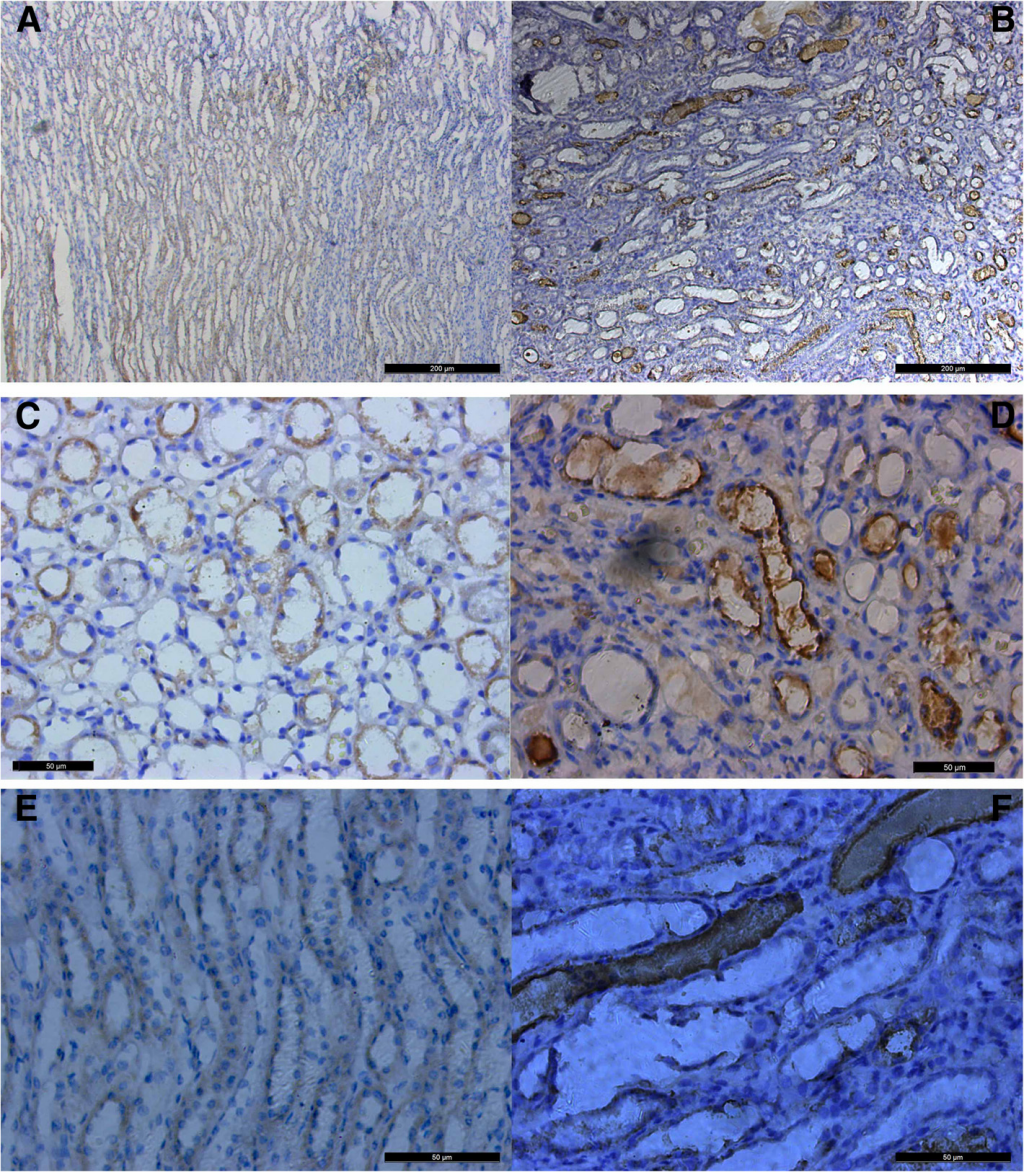
**Changes in protein level of osteopontin in paraffin embedded kidney sections of the treatment group and the controls. A** &**B** expression of OPN in the outer medulla in the TALH and few of collecting ducts in control and treatment groups respectively × 100. **C** &**E** show normal expression of OPN in control group. **D** &**F** show the increased intensity of staining in cellular content and luminal expression of OPN in treatment group. DAB counterstained with hematoxelyne × 400.

Immunohistochemical staining of renal tissue sections revealed different levels of OPN and THP protein expression in the treatment and control dogs. A remarkable increase in the intensity and spread of OPN staining was observed in the tubular lumina as well as in the cells of distal tubules, the medullary thick ascending limbs of the loop of Henle, collecting ducts, in a few number of thin descending limbs of Henle and at the site of inflammation of either cortex or medulla in the kidney of treated dog (Figure 2C & Figure 3). Staining was not detected in the proximal convoluted tubules, glomeruli, or Bowman’s capsules. Staining for OPN was commonly in the cell membrane, cytoplasm of renal epithelial cells, within the tubular lumina and occasionally around crystals. Nuclei were not stained. In the control dogs, epithelial cell staining for OPN was limited to the distal tubules, thick ascending limb of Henle (TALH) and a few collecting ducts.

**Figure 4:**
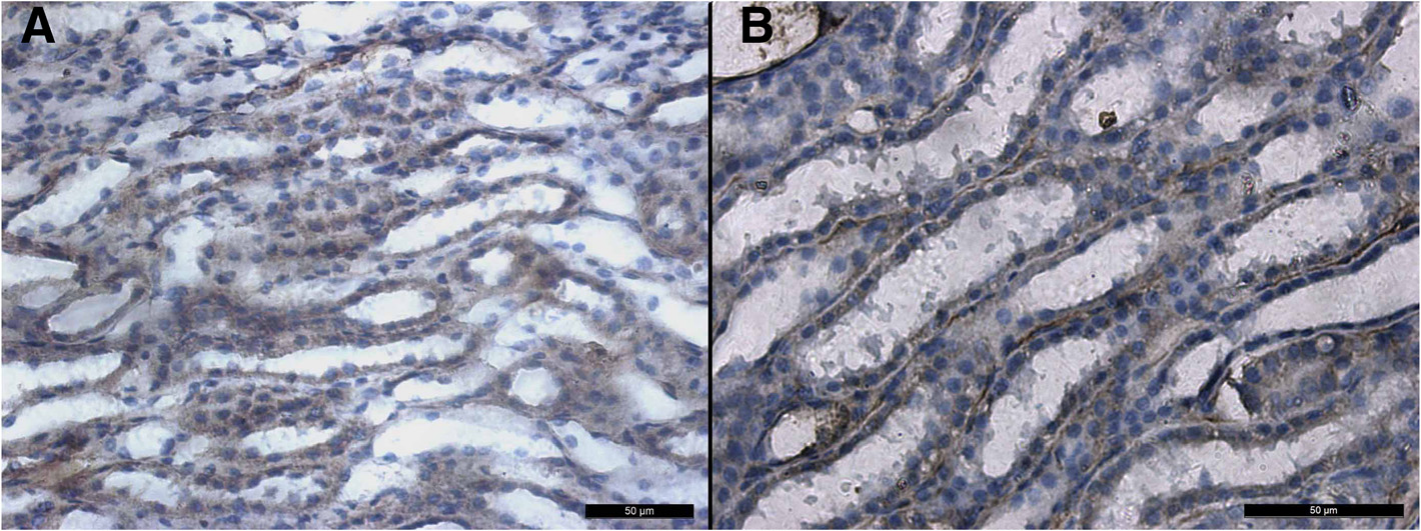
**Changes in protein level of Tamm-horsfall protein in paraffin embedded kidney sections of the treatment group and the controls.** Antigens were detected using antibodies to THP, followed by second antibodies coupled to horseradish peroxidase with DAB counterstained with hematoxelyne**. A)** Expression of THP in control group in TALH. **B)** Decreased expression of THP in treatment group × 400.

Staining for THP in the treatment group decreased compared to the control group in the TALH of the inner cortex and outer medulla. THP was localized at basolateral border. In the control group, staining for THP was restricted to the epithelia of the TALH and some distal convoluted tubules along the apical borders and throughout the cytoplasm. Nuclei were not stained. No other region of the nephron was stained for THP in either the treatment or control group (Figure 4).

### RNA expression of OPN and THP in kidneys

**Figure 5:**
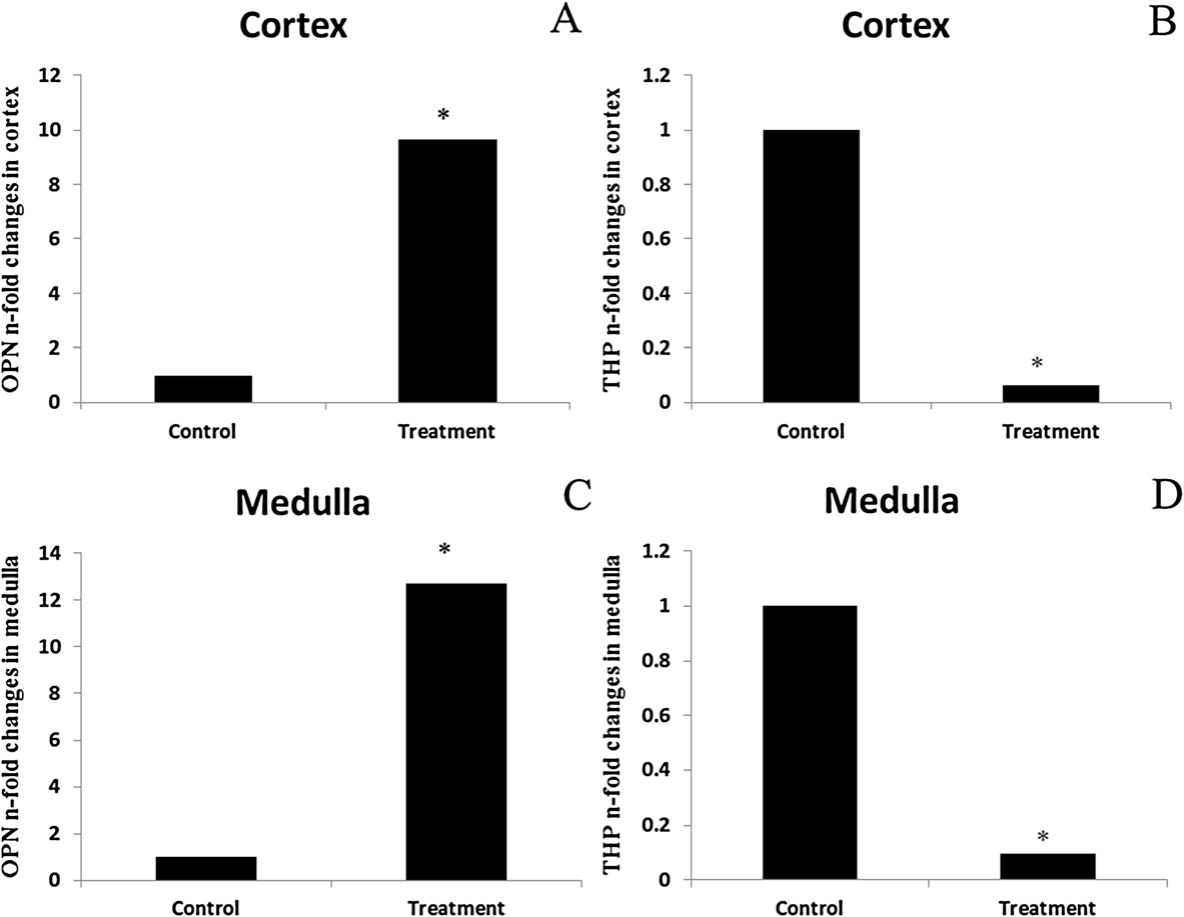
**Fold of change (n-fold) in OPN (A, C) and THP (B, D) mRNA gene expression in cortex and medulla respectively of dogs in each group after 7 days of experiment (*p*** **< 0.01).**

Injection with 0.5 M KOx in treatment group elicited a significant up-regulation of mRNA expression (9.64 folds increase) in cortex and (12.72 folds increase) medulla for OPN (*p* < 0.01) compared to that in the control group (Figure 5A,C). Whereas there was a significant down-regulation of mRNA expression (13.9 fold decrease) and (10.37 fold decrease) in cortex and medulla for THP respectively (*p <* 0.01) compared to that in the control group (Figure 5B,D).

## Discussion

Recent studies indicated that oxalate and CaOx crystals influence the expression of several genes in renal tubular cells which subsequently modify the attachment, growth and aggregation of CaOx crystals [25]. In this study we investigated how oxalate salts and oxalate crystals affected the expression of two proteins produced by the distal regions of the nephron; OPN and THP. These two macromolecules were chosen because their patterns of expression within the nephron are similar but their regulation is different.

OPN is confined to the distal parts of a subset of nephrons. In the kidney the expression of OPN is severely upregulated during renal injury [26]. That might explain why there was an intense staining for OPN indicating a significant increase in OPN protein production in the renal epithelium, tubular lumina and at the sites of inflammation in the treatment group. These results parallel those found in nephrolithiatic rats [21]. The presence of high intensity of OPN staining in the lumen might be attributed to the secretory nature of OPN that, in spite of its high mRNA levels, is being transferred to the extracellular fluid or into secretory products such as urine [27]; moreover crystal retention strongly induces tubular injury and luminal expression of OPN [3].

Data from qPCR demonstrated a significant up-regulation of OPN mRNA in the treatment group. This marked increase in OPN expression can be explained in two ways. One is that crystal/membrane interaction stimulates further OPN mRNA and protein production [22] which enhances crystal adhesion to the renal epithelium and facilitates crystal aggregation which leads to blockage of the tubular lumen. This supports the notion that OPN acts as a stone promoter. But, Grohe et al. [28] considered OPN a promising agent for endogenous stone inhibition by converting CaOx crystallization to the COD phase which is significantly less adhering to renal epithelial cells than the COM phase. This is because COM is more commonly present in kidney stones [29] and COD is more frequently excreted in urine [30]. It is suggested that the structural features of COM crystals may increase their likelihood of being retained within the kidney where they may act as nuclei for stone formation, while those of COD reduce the probability of their attachment to renal cells. Thus, it has been proposed that preferential formation of COD, rather than COM crystals would protect against stone pathogenesis [31]. OPN is one of the urinary proteins bound to the surfaces of COM crystals [32,33] and is also incarcerated within them [34], to inhibit the attachment of COM urinary crystals to renal epithelial cell membranes. Therefore it is possible that the more frequent excretion of the dihydrate might result from differences between individual proteins associated with COM and COD crystals. The last theory was reinforced by studies on OPN knock-out and wild-type mice treated with ethylene glycol (EG). After 4 weeks of treatment with 1% EG, no crystals were retained in wild-type mice, whereas x-ray diffraction analysis of entire tissue sections from OPN knockout mice confirmed that the retained crystals were exclusively COM [35].

Inflammatory cells infiltration is a common feature of tissue injury. The renal damage caused by COM crystal deposition in the kidney may provoke an inflammatory response [36] in which the injured renal tubule cells and macrophages produce various cytokines including OPN [5], which was highly expressed in the renal tissue of the treatment group to attract and retain in the inflammatory cells at the site of inflammation as it coats COM crystals and mediates their attachment to macrophages and interstitial multinucleated cell to facilitate the removal of crystals and tissue repair [37]. But the multifunctional protein, OPN, tasks never end as at the same time it assists in the engulfment of crystals into macrophages and also protects the neighboring healthy tissue from the cytotoxic effect substances such as nitric oxide produced by macrophages [38].

THP, the most abundant protein in urine of humans and animals under physiological conditions, significantly decreased in the urine of stone formers [39]. Although THP is predominantly an apically secreted protein, its abnormal localization during nephropathy has been well-described [40]. In the current study, the immunohistochemistry showed a diminished expression of THP in the treatment group and translocation from the apical membrane and cytoplasm to the basolateral border. qPCR assay revealed a significant decrease in THP mRNA expression level in the treatment group compared to that in the control group, which might be attributed to the toxic effect of oxalates that suppressed THP expression in the renal tissue cells thus promoting COM deposition [18]. Several studies suggested that stone formation could be promoted by quantitative deficiencies in protein synthesis or by molecular abnormalities. These abnormalities may result from defects in primary sequence and/or posttranslational modifications could result in altered conformation and/or patterns of charge density crucial for crystal binding. Dysfunctional molecules could bind less well to crystals or could promote stone formation by other mechanisms such as acceleration of growth kinetics [41] or serving as nucleation sites [42].

Interpretation of OPN results could not be discussed separately from those obtained for THP. We can postulate that after 7 days of oxalate injection renal THP synthesis significantly decreased to encourage crystal growth and/or aggregation around cellular injury. Concurrently, the kidneys promoted OPN up-regulation to minimize crystal deposition and retention. This interpretation was supported by the significantly increased OPN in THP knock-out mice [43]. However, it seemed that any defensive mechanism of OPN was overcome by the COM crystal formation resulting from oxalates exposure.

## Conclusion

For the first time the expression of osteopontin and Tamm-Horsfall protein in canine renal oxalosis were measured. We concluded that the two specific urinary macromolecules OPN and THP secreted by the kidneys responded differently to oxalate injection and participated in an opposite manner in the pathogenesis of semi-acute canine renal oxalosis. In this study we were limited by the scanty literature dealing with these two proteins in canine tissue *in vivo* especially canine OPN, therefore we highlight the need for further studies in the veterinary field about the potential roles of the urinary macromolecules in the pathogenesis of urolithiasis.

## Competing interests

The authors declare that they have no competing interests.
